# Effect of civil war on medical education in Liberia

**DOI:** 10.1186/1865-1380-4-6

**Published:** 2011-02-16

**Authors:** Kathryn R Challoner, Nicolas Forget

**Affiliations:** 1 Faculty, Department of Emergency Medicine Co-Director -International Division of Emergency Medicine Clinical Professor of Emergency Medicine Keck School of Medicine University of Southern California, Los Angeles, USA; 2 Director, Masters Programme in Emergency Medicine Georgetown Public Hospital Corporation Georgetown, Guyana Assistant Professor of Emergency Medicine Vanderbilt University, Nashville, Tennessee, USA

## Abstract

**Background:**

From 1980 to 2003 Liberia entered into a period of conflict and civil wars. During this time Liberia's health and educational services were severely disrupted. Equipment and supplies were stolen from the Medical School and the buildings damaged severely. A majority of health care workers, university faculty, and hospital and medical school administrators fled the country.

**Objective:**

The objective of this study was to evaluate the impact of civil war on the training of medical students and physicians, and to identify a feasible intervention.

**Methods:**

The authors compiled data from three sources at an Emergency Medicine symposium held at the A.M. Dogliotti School of Medicine, in Monrovia, Liberia, in September 2007. These were (1) data from 13 anonymous surveys completed by symposium participants who were physicians or physicians in training, and (2) answers from six open discussion groups at the symposium concerning perceived barriers to medical training. (3) Supporting documents volunteered by the Dean from interviews in 2002, 2007 and 2009 or published on line in 2002 and 2006 were incorporated, and a focused literature review was performed.

**Results:**

The 12 medical students and 1 physician who returned completed surveys and attended the symposium all reported a delay in their training, with 75% of respondents citing a past and current lack of Clinical and Basic Science faculty as a major delaying factor. The six open discussion groups at the symposium and the information provided by the Dean substantiated these findings.

**Conclusions:**

Volunteer Basic Science and Clinical faculty for the medical school and teaching hospitals from a coalition of concerned partnering institutions would be a targeted intervention to assist in re-building the medical educational capacity of Liberia.

## Introduction

The A.M. Dogliotti College of Medicine of Liberia, West Africa, was established in 1968 through a tripartite arrangement involving the Vatican, the Government of Liberia and the Dogliotti Foundation of Italy. The college had a maximum capacity of 125 students and currently has two schools, the College of Medicine and the School of Pharmacy on the College of Medicine campus. This is the only recognized school of medicine in Liberia and has been the major source of Liberia's qualified physicians. Length of training is 5 years plus a 2-year internship. There is no accredited specialty residency training available in Liberia.

From 1980 to 2003, Liberia entered into a period of conflict and civil wars. During this time Liberia's health and educational services were severely disrupted. Many health care workers, university and college faculty, medical school and hospital administrators fled the country. During the war, buildings were badly damaged by armed forces or shell fire, and the contents either stolen or totally destroyed [1].

The war ended, and now the best interventions to help re-build Liberia's medical educational structure needed to be identified and targeted. Our main focus was to obtain as much information as possible on the current status of the Medical School and the barriers to physician training.

## Methods

To evaluate the impact of Liberia's multiple wars on medical education both past and present, we compiled data from three sources.

First we evaluated the data from a survey of participants of a symposium on emergency medicine and trauma care organized at the A.M. Dogliotti College of Medicine of the University of Liberia in Monrovia, Liberia, in September 2007. The symposium was advertised by announcement and posters at the A.M. Dogliotti School of Medicine and JFK Government Hospital in Monrovia. Attendance was recommended to the medical students but was optional as many health care providers could not be given time off from their duties. The participants in the symposium were requested to voluntarily complete an anonymous survey inquiring about (1) demographic information, (2) educational experience to date and (3) the current length of training. They also (4) answered the question, "Has your training been delayed?" If the training had been interrupted or delayed, the respondents were asked to explain using free text answers. Responses were recorded and tabulated (numbers and ranges) on an Excel database.

Second, we collected the collated answers regarding the perceived barriers to academic and clinical training from open afternoon discussion groups with the symposium participants. The format was open discussion while a senior medical student with IRB training on the visiting team took notes and collated the responses.

Third, we summarized information volunteered in interviews by the Dean of the A.M. Dogliotti School of Medicine in 2002, 2007 and 2009, and published online in 2002 and 2006.

### Limitations of these Methods

The symposium was open and free to anyone who could come. Participants therefore consisted of a mix of medical students, physician assistants and nurses, with only a few graduate physicians being available to attend sporadically as staffing at the hospitals was so critical. Attendance also varied from day to day, with some participants only being able to attend part of the symposium's didactic sessions, and consequently these participants did not complete surveys. The survey was optional, and so a convenience sample resulted of participants who stayed for the full symposium and chose to fill out the survey.

## Results

### Surveys

Thirty registered full-time participants at the symposium responded, completed and returned the questionnaire. Only 1 intern and 12 medical students submitted completed surveys, while the rest of the surveys were from nurses, physician assistants and a pharmacist. We primarily focused on the situation of the Medical School and so are reporting the responses of the intern and medical students below, but all the surveys demonstrated that these expressed concerns could be extrapolated to other institutions of higher learning as well.

The average age of the medical students was 36.5 years, and 80% were men. The medical students and intern all reported a delay in training with 83.3% citing the civil war as a cause and 75% citing the lack of Basic Science and Clinical faculty as the major delaying factors.

### Discussion groups

There were six open discussion groups voluntarily attended by 50 Liberian health care professionals including physicians and medical students. The groups targeted the lack of basic science and clinical faculty as both past and current impediments to their training in the Medical School and the teaching hospitals.

They also referenced the extensive damage to the laboratories, library and classrooms of the Medical School during the war and the lack of books, teaching equipment, laboratory equipment and materials needed in training. They mentioned the need for safe nearby dormitory rooms with reliable electricity and water, and the need for a peripheral security fence around the Medical School campus. They discussed the disrepair of the teaching hospitals, closure of other medical facilities, the lack of essential equipment and medication, and the need for improved finances and resources as critical to delivering future medical care to the people of Liberia.

### Supporting documents

In 2002, Tabeh L. Freeman, RN, MD, MPH, Dean of the AM Dogliotti College of Medicine, published a "Situational Analysis" [2] summarizing the current state of the college and its needs. In this document he highlighted the impact of the civil war: "The seven-year civil conflict has caused considerable destruction of the infrastructure of the college, that is, the buildings, library and laboratory facilities, and flight of members of the teaching staff." Most importantly, he stressed that "the shortage of teaching staff was particularly dire." Indeed, with six pre-clinical departments (public health and preventive medicine, anatomy, physiology, biochemistry, pharmacology, and microbiology and parasitology) and seven clinical departments (internal medicine, surgery, radiology, pediatrics, obstetrics and gynecology, pathology and psychiatry), there were four full-time faculty members in the Pre-Clinical Divisions and two full-time faculty in the Clinical Divisions. In the preclinical years, one of the full-time public health teaching staff was the Dean of the Medical School. The Dean wrote that there should be a minimum of one full-time faculty member per department. By those standards, the college needed at least eight more full-time faculty members.

In 2006, Dr. Freeman published an update titled "An Appeal for Assistance" [3]. Per Dr. Freeman's report in 1990, there were over 500 practicing physicians in the country, including the public and private sectors. In 2006 there were fewer than 75, with 26 of those in the public sector [3]. At that time, Dr Freeman reported 34 faculty members at the College--10 full time and 24 part time. He stated that the need for teachers especially in the basic sciences division was the most acute.

In an interview in 2008, Dr. Freeman stated that only 51 Liberian physicians remained practicing in Liberia. Most of A.M. Dogliotti's alumni had fled the country. In 2007 only 4 medical students had graduated (as compared to the usual 40 graduates/year before the civil war), and the average course length had jumped from 5 to 9 years [4].

In 2009, Dean Freeman shared the statistics shown in Table 1 in an interview.

**Table 1:** Teaching faculty at the A.M. Dogliotti School of Medicine in 2009

Pre-Clinical	Full-time teaching staff	Part-time teaching staff	Total
Public Health and Preventive Medicine	5	0	5

Anatomy/Embryology	1	Occasional	1

Histopathology	1	Occasional	1

Physiology	1	0	1

Microbiology and Parasitology	0 - but position filled, professor expected	0	0

Pharmacology	1	0	1

Biochemistry	1	0	1

Total pre-clinical	10	------	10

Clinical	Full-time teaching staff	Part-time teaching staff	Total

Internal Medicine	2	0	2

Surgery	0	0	0

Ob-Gyn	0	0	0

Pediatrics	0	0	0

Psychiatry	0	0	0

Radiology	0	0	0

ENT	1	0	1

Orthopedics	1	1	2

Neurology *	1 on contract	0	1

Anesthesiology *	1 on contract	0	1

Emergency medicine at JFK **	Global Health Alliance -1	0	1

Total clinical	7	1	8

*The Government of Liberia is hiring full-time faculty and staff from other countries on salaried contracts of 1 or more years. The contract of the anesthesiologist from Nigeria will expire in September 2010. ** The Global Health Alliance, partnering with the NGO HEARTT (Health Education and Relief through Teaching), is a consortium of universities dedicated to placing senior residents, fellows and faculty in the Emergency Area of JFK in rotating blocks throughout the year.

The data shows that by 2009, Dean Freeman had recruited at least one basic science faculty member to every department. However, there still remained a clear need for more full-time clinical faculty in every department of the Medical School and teaching hospitals. Part-time and visiting professors are also critical in supplementing the gaps when full-time professors are on needed leave.

The Dean also stressed that just when there was a compelling need to graduate more and more qualified physicians, the institution qualified to do so was reporting an on-going lack of teaching faculty.

## Discussion

The prolonged civil conflict caused major damage to the health care system of Liberia [5]. The medical students were severely impacted, with 100% reporting a delay in training, and 83.3% of them citing the civil war and 75% citing a lack of clinical and basic science faculty as delaying factors in the surveys.

In the discussion groups the students further defined the impact of the civil war on training delays due to loss of laboratory, library, teaching and residential facilities, destruction of the infrastructure and lack of personal security.

However, since 2007, because of a grant from the World Bank and other generous donations, a security fence for the Medical School has been built, and laboratories, classrooms and administrative offices are being constructed. The country is now at peace.

The greatest need both past and present is for new faculty members-especially Clinical, but also Basic Science-to allow the A.M. Dogliotti Medical School to graduate the number of qualified physicians necessary to re-build Liberia's medical delivery system.

## Conclusion

An identified and important targeted intervention includes volunteer or subsidized Basic Science and Clinical faculty members for the Medical School and teaching hospitals from a coalition of concerned partnering institutions to assist in re-building the medical educational capacity of Liberia. The long-term rewards of this would be immeasurable-a country's health-care infrastructure restored and Liberian physicians trained to deliver health care in a West Africa country recovering from a horrible and crippling civil war.

## Competing interests

KC has no competing interests.

NF has no competing interests.

## Authors' contributions

NF compiled and summarized the data and references, and wrote the methods and results sections. NF has read and approved the final manuscript. KC did the literature search and the interviews and wrote the abstract, introduction, discussion and conclusions sections of the paper. KC has read and approved the final manuscript.
